# Tuberculosis as a marker of inequities in the context of socio-spatial transformation

**DOI:** 10.1590/S1518-8787.2017051006533

**Published:** 2017-02-08

**Authors:** Alexandre San Pedro, Gerusa Gibson, Jefferson Pereira Caldas dos Santos, Luciano Medeiros de Toledo, Paulo Chagastelles Sabroza, Rosely Magalhães de Oliveira

**Affiliations:** I Departamento de Endemias Samuel Pessoa. Escola Nacional de Saúde Pública. Fundação Oswaldo Cruz. Rio de Janeiro, RJ, Brasil; II Instituto de Estudos em Saúde Coletiva. Universidade Federal do Rio de Janeiro. Rio de Janeiro, RJ, Brasil

**Keywords:** Tuberculosis, epidemiology, Socioeconomic Factors, Health Inequalities, Social Inequity, Ecological Studies

## Abstract

**OBJECTIVE:**

This study aims to analyze the association between the incidence of tuberculosis and different socioeconomic indicators in a territory of intense transformation of the urban space.

**METHODS:**

This is an ecological study, whose analysis units were the neighborhoods of the city of Itaboraí, state of Rio de Janeiro, Brazil. The data have been analyzed by generalized linear models. The response variable was incidence of tuberculosis from 2006 to 2011. The independent variables were the socio-demographic indicators. The spatial distribution of tuberculosis was analyzed with the elaboration of thematic maps.

**RESULTS:**

The results have shown a significant association between the incidence of tuberculosis and variables that reflect different dimensions of living conditions, such as consumer goods, housing conditions and its surroundings, agglomeration of population, and income distribution.

**CONCLUSIONS:**

The disproportionate incidence of tuberculosis in populations with worse living conditions highlights the persistence of socioeconomic determinants in the reproduction of the disease. Different municipal public sectors need to better articulate with local tuberculosis control programs to reduce the social burden of the disease.

## RESUMO

**OBJETIVO:**

Analisar a associação entre incidência de tuberculose e diferentes indicadores socioeconômicos em território em intensa transformação do espaço urbano.

**MÉTODOS:**

Estudo ecológico, cujas unidades de análise foram os bairros do município de Itaboraí, RJ. Os dados foram analisados por modelos lineares generalizados. A variável resposta foi incidência de tuberculose de 2006 a 2011. As variáveis independentes foram os indicadores sociodemográficos. A distribuição espacial da tuberculose foi analisada por meio da elaboração de mapas temáticos.

**RESULTADOS:**

Os resultados apontaram associação significativa entre a incidência de tuberculose e variáveis que refletem diferentes dimensões de condições de vida, como bens de consumo, condições de moradia e seu entorno, aglomeração populacional e distribuição de renda.

**CONCLUSÕES:**

O acometimento desproporcional da tuberculose em grupos populacionais com piores condições de vida ressalta a persistência dos determinantes socioeconômicos na reprodução da doença. Faz-se necessária a melhor articulação de diferentes setores públicos municipais com o programa local de controle da tuberculose visando à redução da carga social da doença.

## INTRODUCTION

Among the individual and contextual determinants related to tuberculosis, socioeconomic conditions are seen as important factors associated with the reproduction of the disease^4,9,12,15,18^. Tuberculosis operates as a marker of social inequities in health linked to precarious living conditions. That is why the World Health Organization has listed the reduction of the socioeconomic burden of the disease as one of the most important goals to be achieved until 2015^a^.

However, the focus of programmatic activities that are predominantly fixated on the identification and timely treatment of cases, as well as the lack of dialog with other government sectors, limits the scope of municipal control programs for the reduction of the social burden of the disease. Such a situation can be exacerbated in territories undergoing an important process of transformation of urban space induced by the implementation of major economic development projects. The expectation of increased land values, increased economic inequalities and deepened social division, in addition to other factors such as the arrival of a migrant population with a low degree of technical training and little prospect of integration into the formal economy circuit^1^, can exert strong influence on the reproduction of tuberculosis.

The city of Itaboraí, State of Rio de Janeiro, Brazil, has a history of high incidence of tuberculosis and it is undergoing important transformations of its urban space to host the largest petrochemical complex in the Latin America. The city suffers significant transformations in its urbanization pattern since the beginning of the implementation of the project in 2008, with an increase in housing informality and in the precariousness of housing conditions arising from deprivation of environmental sanitation^b^. The number of households in precarious settlements increased 79.0% between 2000 and 2010, concurrently with the increase in land values since the enunciation of the implementation of the project in the city^b,c^.

The rapid process of socio-spatial reorganization of the region results in the formation of conflicting territorial arrangements in which urban patterns with appropriate infrastructure and services live next to spaces marked by housing informality and precarious urban infrastructure^1^. This demand a high adaptive and analytical capacity from local control programs regarding the ongoing changes in the epidemiological situation of the disease.

The importance of socioeconomic factors related to the process of reproduction of tuberculosis in intra-urban agglomerations is known^5,15,19^. Nevertheless, little progress has been made in the discussion of socio-spatial patterns related to the evolution of the disease in contexts under intense transformation, as in Itaboraí. This study has aimed to analyze the relationship between the occurrence of tuberculosis and different socioeconomic dimensions linked to the living conditions of an endemic city undergoing a process of transformation of its urban space.

## METHODS

This is an ecological study, whose analysis units were the seventy-nine neighborhoods of Itaboraí. The city is located in the region *Leste Fluminense*, 46 km from the city of Rio de Janeiro. The association between the incidence of tuberculosis from 2006 to 2011 and the socioeconomic indicators related to the neighborhoods of the city were analyzed by generalized linear models.

Historically, Itaboraí is characterized by its importance in the economic dynamics of the region in which it is inserted. In the colonial period, it was considered the main trading post of the sugar production of Eastern *Baixada da Guanabara*, a place of convergence of herdsmen transporting the sugar and coffee production from neighboring regions to the city of Rio de Janeiro^d^. Currently, it has strategic importance for the economic development of the State of Rio de Janeiro and even Brazil, as it hosts the largest petrochemical industrial complex of Latin America. With the beginning of the implementation of the Petrochemical Complex of Rio de Janeiro (COMPERJ) in 2008, the city started experiencing a significant transformation of its urban space. Economic activities proliferated, aimed at the service and real estate speculation sectors, with expectations of increased migratory flow after the implantation of downstream petroleum industries.

Itaboraí had a census population of 218,000 inhabitants in 2010, with its Municipal Human Development Index (MHDI) being considered as intermediate (0.69), in addition to a monthly average income of R$1,860, and Gini index reflecting reasonable inequality of income distribution (0.48)^e,f^.

The tuberculosis control program has been decentralized since 2003. That year, the strategy *Tratamento Diretamente Observado* (TDO – Directly Observed Treatment) was implemented in thirty-four Family Health Units (FHU), responsible for forty-five teams of the Family Health Strategy (FHS), whose percentage of territorial coverage was approximately 77.0%^g^. The strategy of the TDO was extended to patients not covered by the FHS in 2005, and important results were achieved in the five-year period, such as the reduction from 30.0% to 5.0% in the abandonment of the treatment and increase from 60.0% to 88.0% in the percentage of cases cured^16^. Despite these important results, Itaboraí is one of the 181 priority Brazilian cities for the control of the disease as it presents incidence rates above 80.0% of the estimated rate for the country (32 new cases /100,000 inhabitants)^6^ since 2007.

The study sample consisted of new cases of pulmonary tuberculosis registered in the *Sistema Nacional de Agravos de Notificação* (SINAN – Notifiable Diseases Information System) from 2006 to 2011. We organized the cases according to the neighborhood of residence. For the records that had no information about the neighborhood, we crossed the street address with the registration of streets and land developments of the neighborhoods provided by the Local Government, resulting in completeness of 93.4% for this variable. We obtained the socioeconomic data used for the construction of indicators from the 2010 Census of the Brazilian Institute of Geography and Statistics (IBGE) and aggregates for the territorial level of neighborhoods. We based the population estimates for the intercensal period on the geometric growth model, assuming constant linear variation per unit of time (year).

The criteria for the initial selection of indicators considered the importance in determining the production and living conditions of tuberculosis, based on a study of systematic review^15^ on the topic, as well as the availability of data in the 2010 Census. We did not seek the directionality of the indicators *a priori*; on the contrary, we wanted a broader analysis of the relationship between socioeconomic inequality and occurrence of tuberculosis. We constructed indicators grouping them according to different dimensions of living conditions (Table 1).

**Table 1:** Selected socioeconomic variables according to different dimensions of living conditions.

Dimensions	Variables	Description
Consumer goods	Private vehicle	Percentage of households with private vehicle
	Microcomputer	Percentage of households with microcomputer
Health infrastructure	Supply of Water	Percentage of households connected to the general network of water supply and indoor plumbing in at least one room
	Sewage system	Percentage of households connected to the general network of rainwater or sewage system
Housing conditions and surroundings	Coating of exterior walls	Percentage of households without coating on the external walls
	Identification of the street address	Percentage of households without identification of street address
	Open sewer	Percentage of households with open sewer in the surroundings
	Street lighting	Percentage of households without street lighting in the surroundings
	Accumulated waste	Percentage of households with accumulated waste in the surroundings
Income	Proportion of poor persons	Percentage of heads of household with income up to one minimum wage
	Theil Index	Inequality in income distribution of the heads of household^a^
Education	Education level	Percentage of heads of household with incomplete High School
Social assistance	Social security contribution	Percentage of taxpayers with official social security in their main job or in another job
	Government financial benefits	Percentage of beneficiaries of the Social Program *Bolsa Familia* or *Programa de Erradicação de Trabalho Infantil* (Program on the Elimination of Child Labor)
Population agglomeration	Population density	Number of inhabitants according to total usable area by km^2b^
	Density of the poor	Number of heads of domicile with income up to one minimum wage according to total usable area by km^2^
	Density per room	Percentage of households with more than three residents per room
Migration	New immigrants	Percentage of persons who immigrated between one and two years before the 2010 Census
	Old immigrants	Percentage of persons who immigrated more than ten years before the 2010 Census

^a^ Souza PFL, Salvato MA. Decomposição hierárquica da desigualdade de renda brasileira. In: XXXVI Encontro Nacional de Economia. 2008, Salvador – Bahia. Anais do XXXVI Encontro Nacional de Economia. 2008. p.1-21. Available from: http://www.anpec.org.br/encontro2008/artigos/200807211123470-.pdf

^b^ Buildable portion of the neighborhood, estimated by the merged image of the sensors PRISM (Panchromatic Remote-sensing Instrument for Stereo Mapping) and AVINIR (Advanced Visible and Near Infrared Radiometer type 2) of the satellite ALOS (Advanced Land Observing Satellite).

The response variable was the number of cases of pulmonary tuberculosis according to the neighborhoods of Itaboraí between 2006 and 2011. We analyzed the data by generalized linear model (GLM) with Poisson distribution. However, we decided to use GLM with negative binomial distribution because of the great dispersion of the multiple final model^8^.

The model used assumes the form log (µ) = βx (log link function), where x_i_ is the explanatory variable. The population was included as offset level considering its logarithm of each neighborhood. The exponential of regression coefficients β_i_ represents the Incidence Rate Ratio (IRR)^9^. We tested the explanatory variables separately. Those that showed statistical significance (p < 0.20) were included, one by one, in the negative binomial regression with log link function (forward method). Before the analysis, we tested for multicollinearity using VIF (Variance Inflation Factor) among the independent variables, with tolerance of less than ten. We selected the multiple model with best fit by the likelihood ratio test^8^, which consists of a hypothesis test that compares the quality of fit between two models, in which one of the models is the subset of the other (nested models). We used Moran’s I index for spatial autocorrelation in the residuals from the final multiple model.

We carried out a descriptive analysis of the spatial distribution of tuberculosis from thematic maps of the average incidence by neighborhoods for the times related to the period of enunciation and beginning of works (2006 to 2008) and the period in which the installation process of the projects was intensified (2009 to 2011). To compare the periods, we divided the classes by quartile from the set of rates (2006 to 2011), which were smoothed by the Empirical Bayes method, assuming a matrix of neighborhood by contiguity. We decided to smooth in this step because of the neighborhoods with small population, as they result in greater instability of the estimated gross rates.

This study was carried out according to the determinations of the Research Ethics Committee of the Escola Nacional de Saúde Pública Sergio Arouca (Opinion 71,237/2012).

## RESULTS

Eight hundred and four new cases of pulmonary tuberculosis were reported in Itaboraí from 2006 to 2011, with an annual average of 134 cases (min. = 103, max = 158) and average annual rate of incidence of 64.4 cases per 100,000 inhabitants. Table 2 presents the measures of central tendency and dispersion of the indicators according to the neighborhoods of Itaboraí.

**Table 2:** Measure of central tendency (arithmetic mean) and dispersion of socioeconomic indicators pre-selected in the univariate analysis, according to neighborhoods. Itaboraí, RJ, Southeastern Brazil, 2010.

Dimensions of living conditions and socioeconomic indicators	Mean	Standard deviation	Min.-Max.
Consumer goods			
Households with microcomputer	29.9	11.3	7.8-66.0
Households with private vehicle	29.3	12.1	6.1-58.0
Housing conditions			
Households without coating on the external walls	19.1	10.8	0-42.8
Households connected to the general sewage network	27.6	26.1	5.05-84.7
Households without identification of street address	39.8	21.4	2.7-85.4
Households with open sewer in the surroundings	16.1	14.5	0-37.8
Social assistance			
Social security taxpayers	60.6	11.4	33.7-92.3
Beneficiaries of government cash transfer programs (*Bolsa Família* or Program on the Elimination of Child Labor)	6.9	3.5	0-15.7
Income distribution			
Theil Index	0.4	0.1	0.2-0.6
Density			
Density of the poor	292	271.2	20–1,084
Population density	1,834.0	1,775.8	102–6,050
Households with more than three residents per room	9.1	4.6	1.0-21.3
Migration			
Migrant between one and two years before the Census	4.9	2.6	0-11.6
Migrant for more than ten years before the Census	28.3	10.1	7.3-50.9

Note: The socioeconomic variables have been expressed as percentage.

Regarding the spatial distribution of the disease, neighborhoods with higher rates in the first period were located in the two main urban axes of the city (Southwest and Center). On the other hand, the second period (2009 to 2011) suggested a reduction of rates in the neighborhoods located in these urban axes, but with persistence in some neighborhoods with higher densities of poor persons (Figure – A, B, C). The proximity to the main highways that go through Itaboraí was a common characteristic between the territorial units with higher rates of the disease in both periods analyzed.

**Figure:**
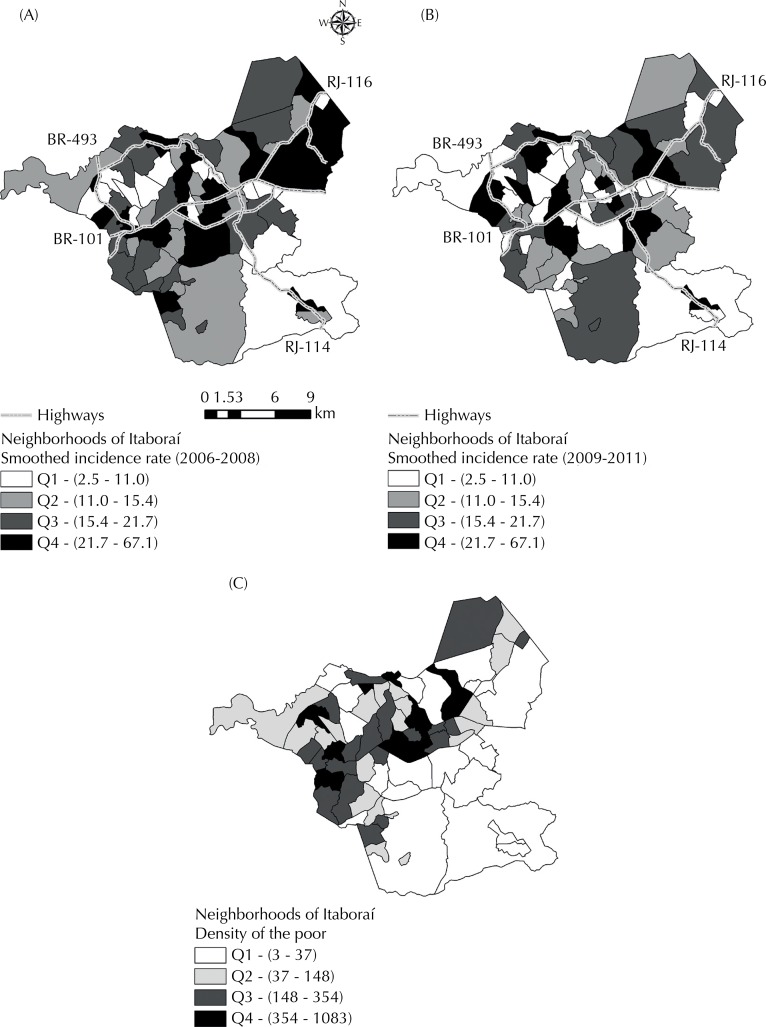
(A) Spatial distribution of tuberculosis according to neighborhoods. Itaboraí, RJ, Southeastern Brazil, from 2006 to 2008; (B) Spatial distribution of tuberculosis according to neighborhoods. Itaboraí, RJ, Southeastern Brazil, from 2009 to 2011; (C) Spatial distribution of the indicator “density of the poor” according to neighborhoods. Itaboraí, RJ, Southeastern Brazil, 2010.

The univariate analysis between incidence of tuberculosis and socioeconomic indicators pointed to significant association with variables related to living conditions (Table 3).

**Table 3:** Parameters of the univariate analysis and multiple model with the respective tuberculosis incidence rate ratios according to neighborhoods. Itaboraí, RJ, Southeastern Brazil, 2006 to 2011.

Dimensions of living conditions and socioeconomic indicators	IRR	95%CI	p
Consumer goods			
Households with microcomputer	0.73	0.55–0.98	0.03
Households with private vehicle	0.63	0.49–0.82	0.00
Availability of health infrastructure			
Households connected to the general sewage network	1.64	1.48–1.81	0.00
Housing conditions and surroundings			
Households without coating on the external walls	1.82	1.56–2.13	0.00
Households without identification of street address	1.25	1.08–1.45	0.00
Households with open sewer in the surroundings	1.51	1.32–1.73	0.00
Social assistance			
Social security taxpayers	0.43	0.20–0.89	0.00
Beneficiaries of government cash transfer programs (*Bolsa Família* or Program on the Elimination of Child Labor)	2.20	1.68–2.86	0.00
Income distribution			
Theil Index	1.95	1.06–3.60	0.03
Population agglomeration			
Density of the poor	1.26	1.13–1.40	0.02
Population density	1.24	1.12–1.38	0.00
Households with more than three residents per room	1.69	1.35–2.09	0.00
Migration			
Migrant between one and two years before the Census	1.50	1.21–1.86	0.00
Migrant for more than ten years before the Census	0.75	0.53–1.07	0.11
Multiple model			
Households with private vehicles	0.46	0.35–0.60	0.00
Households with more than three residents per room	1.39	1.10–1.76	0.00
Households connected to the general sewage network	1.29	1.96–2.66	0.00
Households with open sewer in the surroundings	1.42	1.22–1.66	0.00
Density of the poor	1.20	1.08–1.34	0.00
Migrant between one and two years before the Census	1.44	1.17–1.78	0.00
Theil Index	1.98	1.05–3.73	0.00

IRR: Incidence Rate Ratio

We have observed inverse associations between incidence of tuberculosis and the variables related to consumer goods and direct associations with variables related to housing conditions and surroundings and appropriate infrastructure (Table 3).

We have observed inverse association between incidence of tuberculosis and percentage of social security taxpayers and direct association with percentage of beneficiaries of income transfer programs. The greater inequality of income distribution, measured by the Theil index, was directly associated with occurrence of tuberculosis (Table 3).

We have also observed direct association with recent migration and population agglomeration in the territory and density per room (Table 3).

The model with best fit in the multiple analysis had these variables: percentage of households with private vehicle, percentage of households with more than three residents per room, percentage of households with presence of open sewer in the surroundings, density of the poor, Theil index and percentage of recent migrants (Table 3).

Moran’s I index, related to the residuals of the final multiple model, showed no statistical significance (p = 0.483), thus indicating the absence of spatial autocorrelation.

## DISCUSSION

The remarkable relationship between the occurrence of tuberculosis and socioeconomic inequalities observed in this study highlights an important framework of social inequities in health, evidenced not only in our results, but also in other studies using different territorial units^3,7,10,13^.

The pattern of spatial distribution of tuberculosis for the study area can be the result of a socio-spatial reorganization, with the displacement of the low-income population to areas that lack health and housing infrastructure because of the increased real estate speculation^b,c^. The limits and scope of the local control program to reduce the rates in these new urban agglomerations in socioeconomic vulnerability situation should be assessed.

The higher incidence of tuberculosis in neighborhoods with higher coverage of the general sewage network in the study area suggests the existence of socio-spatial heterogeneity because of the presence of pockets of poorer populations in neighborhoods with better health infrastructure. Such aspect is reinforced by the higher incidence of tuberculosis in neighborhoods with greater inequality of income distribution (Theil index). This information points to the need of studies to be developed at the local level that can identify particular characteristics not captured or included in analyses of higher level of spatial aggregation^14^.

Although we have identified higher incidences of tuberculosis in neighborhoods with higher population density and higher density per room, these indicators do not always relate with the disease directly. The high concentration of persons in a given space does not necessarily mean poor living conditions, as discussed by Vicentin et al.^18^ (2002). The density of the poor proved to be more suitable as the marker of these spaces, as it aggregates individual and territorial attributes, indirectly measuring the rate of social contact from the densification of persons who live in situations of insecurity and who are less able to act/respond to tuberculosis^2^.

The higher incidence of tuberculosis in neighborhoods with higher percentage of beneficiaries of government cash transfer programs (*Bolsa Família* and Program on the Elimination of Child Labor) suggests greater vulnerability because of the poverty or extreme poverty present in these areas.

Studies show positive effects of cash transfer programs on the health situation, with significant impacts on the reduction of inequality of income distribution in Brazil from 2000, as a result of the economic and social inclusion of families living in poverty^17,h,i^.

Although they grant improved nutritional status and greater access to health services^11^, in this sectional study the highest incidence of tuberculosis in neighborhoods with higher proportions of beneficiaries of these programs is present as a marker of poor living conditions and, ultimately, of the greater vulnerability to tuberculosis. For a better understanding of the impact of these programs on the occurrence of tuberculosis, we need to consider the percentage of coverage, time of implementation and interaction of those programs with the Family Health Strategy, as emphasized by Rosela et al.^17^ (2013).

The contribution of recent residents is estimated to be approximately 4.4% of the city population, comprising persons in economically active age (15 to 59 years). Although the contribution of immigrants to this city is not very intense, its occurrence suggests the influence of COMPERJ as an attractive hub and vector of organization of the territory. At the time of the initial implementation of the project, migration flows were low. However, a more significant contribution of immigrants may happen with the beginning of the operation of the refinery and installation of downstream petroleum industries, as in the city of Macaé, RJ, which experienced a process similar to what is happening in Itaboraí^j^.

The higher incidence of tuberculosis in neighborhoods with higher proportion of recent immigrants is an important challenge to the disease control program of the city. The arrival of immigrants with low degree of technical training and little prospect of integration into the formal economy circuit results in the occupation of areas with worse housing conditions and reduced public services. These socio-spatial patterns imply a greater risk of exposure to tuberculosis. The great challenge of the local control program is the development and consolidation of a surveillance that can identify new urban spaces vulnerable to the disease.

The use of the neighborhood as unit of analysis could capture the relationship between socioeconomic inequalities and tuberculosis. However, the internal heterogeneity of these territorial units could not discriminate the presence of pockets of worse living conditions where actions to control tuberculosis could be intensified. Considering the relevance of the service to control tuberculosis, another limitation that could not be controlled in this study is the unavailability of an indicator that would measure the quality of the care of the FHS in each neighborhood, as the good coverage in the city does not necessarily mean adequate assistance to the cases of tuberculosis.

Our results corroborate the findings in other studies regarding the importance of socioeconomic determinants for tuberculosis, showing the disproportionate incidence of the disease among population groups with worse living conditions^7,16,19^.

The implementation of an important vector of economic development in the study area provides a set of opportunities, stresses and vulnerabilities experienced in a rapidly changing territory. On the one hand, the implementation of this type of project generates opportunities for the Government to expand and improve housing, health, and education services because of the collection of taxes and royalties. On the other hand, the increase in socioeconomic inequalities and real estate speculation raises tensions and vulnerabilities related to the unequal appropriation of the territory, in which those with little economic power occupy neighborhoods with little public investment in housing infrastructure.

The collaboration of health services and other municipal public sectors focused on housing, infrastructure, social welfare, and education is very important to reduce the social burden of tuberculosis so that the problem is not solved solely based on the responsiveness of local disease control programs.

## Footnotes

a World Health Organization. Tuberculosis (TB): The stop Tb strategy. Geneva: World Health Organization; 2006 [ cited 2015 Oct 20]. Available from: http://www.who.int/tb/strategy/en/

b Freire EBH, Oliveira NC, Bienenstein R. Reconfiguração territorial em tempo de grandes projetos regionais: o caso do Leste Metropolitano do Rio de Janeiro. In: Anais do 15º Encontro Nacional da ANPUR; 2013; Recife. p. 1-17.

c Silva TB, Lima SF. Políticas públicas habitacionais no município de Itaboraí: um estudo de novos investimentos. In: Crises, práxis e autonomia: espaços de resistência e de esperanças: espaço de diálogos e práticas. Anais do 16º Encontro Nacional dos Geógrafos; 25-31 jul 2010. p. 1-11.

d Machado LO. Diagnóstico socioeconômico da Bacia do Caceribu: gente de Caceribu, sua geografia, sua história. 1998 [cited 2015 Oct 20]. Available from: http://acd.ufrj.br/fronteiras/trabtec/caceribu.htm

e Instituto Brasileiro de Geografia e Estatística. Censo 2010. Rio de Janeiro: IBGE [cited 2015 Oct 20]. Available from: http://www.censo2010.ibge.gov.br/resultados

f Programas das Nações Unidas para o Desenvolvimento. Atlas de desenvolvimento humano no Brasil. 2013 [cited 2015 Oct 20]. Available from em: http://www.pnud.org.br/IDH/Atlas2013.aspx?indiceAccordion=1&li=li_Atlas2013

g Barbosa SQ. As práticas educativas em saúde relacionadas à tuberculose: estudo de caso em uma unidade de saúde da família no município de Itaboraí [dissertation]. Rio de Janeiro (RJ): Escola Nacional de Saúde Pública, Fundação Oswaldo Cruz; 2012.

j Terra D, Ressiguier JH. Mudanças no espaço urbano de Macaé: 1970-2010: Impactos sociais, ambientais e urbanos das atividades petrolíferas: o caso de Macaé. 2010; capítulo 2; p.149 [cited 2015 Oct 20]. Available from: http://www.uff.br/macaeimpacto/OFICINAMACAE/pdf/AZ_ OficinaImpactosMacae TodosTextos.pdf
